# Influence of the Toothpaste with Brazilian Ethanol Extract Propolis on the Oral Cavity Health

**DOI:** 10.1155/2013/215391

**Published:** 2013-06-04

**Authors:** Dariusz Skaba, Tadeusz Morawiec, Marta Tanasiewicz, Anna Mertas, Elżbieta Bobela, Ewelina Szliszka, Małgorzata Skucha-Nowak, Monika Dawiec, Rindai Yamamoto, Shinobu Ishiai, Yuki Makita, Małgorzata Redzynia, Beata Janoszka, Iwona Niedzielska, Wojciech Król

**Affiliations:** ^1^Department of Conservative Dentistry with Endodontics, Faculty of Medicine and Dentistry, Medical University of Silesia in Katowice, Plac Akademicki 17, 41902 Bytom, Poland; ^2^Department of Oral Surgery, Faculty of Medicine and Dentistry, Medical University of Silesia in Katowice, Plac Akademicki 17, 41-902 Bytom, Poland; ^3^Department of Microbiology and Immunology, Faculty of Medicine and Dentistry, Medical University of Silesia in Katowice, Ul Jordana 19, 41-808 Zabrze, Poland; ^4^Nihon Natural Therapy Research Laboratory, 6-26-12 Nishishinjuku, Shinjuku-ku, 160-0023 Tokyo, Japan; ^5^Nippon Zettoc Research Laboratory, 3-26 Kudan-Minami 2-Chome, Chiyoda-ku, 102-0074 Tokyo, Japan; ^6^Institute of Technical Biochemistry, Faculty of Biotechnology and Food Sciences, Lodz Technical University Ul Stefanowskiego 4/10, 90-924 Łódź, Poland; ^7^Department of Chemistry, Faculty of Medicine and Dentistry, Medical University of Silesia in Katowice, Ul Jordana 19, 41-808 Zabrze, Poland

## Abstract

Propolis-based therapeutic agents represent this potential for the development of new drugs in dental care. The aim of a clinical-cohort study was to determine the influence of application of toothpaste enriched with Brazilian extract of propolis (EEP) on health status of oral cavity. Laboratory analysis was conducted in order to assess the chemical composition of EEP including total phenolic compounds, the DPPH (1,1-diphenyl-2-picrylhydrazyl) radical scavenging activity, ABTS radical cation scavenging activity, and FRAP assay. Clinical research involved two groups of subjects comprising 32 adult patients, with assessment based on the preliminary evaluation of the state of their marginal periodontium. The investigation of oral health indices API, OHI, and SBI and microbiological examination of oral microflora were also carried out. Results obtained indicated time-dependent microbial action of EEP at 50 mg/L concentration, with antimicrobial activity against Gram-positive bacteria. The total decrease of API, OHI, and SBI mean values was observed. Hygienic preparations with 3% content of Brazilian ethanol extract of green propolis (EEP) efficiently support removal of dental plaque and improve the state of marginal periodontium.

## 1. Introduction

Gingivitis and periodontitis, apart dental caries, are the most common diseases of the oral cavity. Bacteria existing in the dental plaque are the major etiologic factor of marginal periodontitis.

Periodontitis is a chronic inflammatory disease caused by oral bacterial infection. Imbalance between the initiation of bacterial pathogens and the host immune response to infection contributes to the imitation or progression of periodontitis [1].

 Moreover, there are many general and local factors modifying the onset and the course of the disease. One of the most important measures to be undertaken in order to fight gingivitis and periodontitis is maintenance of proper hygiene of the oral cavity. Choosing an adequate method of teeth brushing, a proper toothbrush and supporting products are the first step in the fight with the disease. The research to improve the content of toothpastes and mouthwashes has been continuing for many years so that, besides better abrasive and polishing properties, they should become better in terms of therapeutic abilities. One of the known methods to improve them is addition of natural ingredients (e.g., ethanol extract of propolis) which are successfully used in other areas of medicine as substances that accelerate the wound healing process [1]. 

Plant products have been used since ancient times in folk medicine, involving both Eastern and Western medical traditions over the past decades. Pharmaceutical companies have been interested in investigating plants as sources for new therapeutic agents with proven efficacy, safety, and quality [2–10]. Propolis has been used in dentistry for various purposes and has a promising role in future medicine as well as in dentistry.

The present study has addressed many samples of propolis with potential source for new therapies in dentistry [11]. In the last decades, several works dealing with propolis composition and biological properties have been published, revealing the interest of researchers on this bee product and its potential for the development of new drugs as well.

Propolis can be used in the management of dental caries and endodontic as well as periodontal infections, vital pulp therapy and in the treatment of oral lesions and repair of surgical wounds [12–17].

Propolis (bee glue) is a sticky dark-colored material that honey bees collect from plants and use in the hive: they apply it to seal the walls, to strengthen the borders of combs, and to embalm dead invaders [18]. Because of its popularity in folk medicine, propolis has become the subject of intense pharmacological and chemical studies for the last 40 years [18]. Numerous studies have proven its versatile pharmacological activities: antibacterial [19, 20], antifungal [21, 22], antiviral [23], antiinflammatory [24–26], antitumor [27], antioxidant and “free radical scavenger” [28, 29], as well as immunomodulatory action [30–32], radioprotective [33], and so forth. [34–37]. Chemically, propolis of different parts of the world is constituted by 50–60% of resin, 30–40% of wax, 5–10% of essential oils, 5% of pollen, besides microelements [18]. So far, more than three hundred organic compounds of different groups mainly phenolic, such as flavonoids, stilbenes, phenolic acids, and their esters, have been identified from propolis [18, 38, 39]. Moreover, this class of compounds are responsible for many of the biological activities attributed to European propolis [39–46].

It is well accepted that the chemical constituents and health properties of propolis greatly depend on several ecological factors, including geographical region, plant source, season, and method of harvesting [38, 47–49]. European, Chinese, and Argentinean propolis are characterized by the presence of phenolic acid and flavonoids and the most abundant were chrysin (2–4%), pinocembrin (2–4%), pinobanksin acetate (1.6–3%), and galangin (1-2%). Some Brazilian propolis contains mainly artepillin C, different caffeoylquinic acids and some flavonoids [50].

Several studies on the chemistry and biological activities of Brazylian propolis have previously been reported [13, 14, 16, 22, 26, 27, 47, 51–54]. Brazilian propolis was classified into 12 groups based on the physicochemical characteristics [47]. The most commercialized propolis type is known as “green propolis” and it has been extensively studied and used in food and beverages. However, red propolis has been an important source of investigation since 2007 by research groups [54, 55]. The botanical origin of propolis group 12 was the resin of *Baccharis dracunculifolia* in Southeast Brazil [56, 57].

The aim of this work was to determine and investigate the influence of application of toothpaste with EEP (specimen T) on status of oral cavity, in comparison with the same kind of toothpaste without EEP (specimen G). Sample of propolis was collected from Southeast Brazil (green propolis).

## 2. Materials and Methods

### 2.1. Propolis

Raw propolis was collected from the beekeeping section of the Seiri Alimentos Naturais Brazil.

Propolis samples were obtained from colonies of Africanized honeybees (*Apis mellifera*) in Minas Gerais State, Southeast Brazil, and collected using plastic net.

The unprocessed propolis was sent to the Nihon Natural Therapy Co., Ltd., Tokyo, Japan, for preparation of the EEP. The toothpaste with 3% of EEP and without EEP (placebo) was prepared in Nippon Zettoc Co., Ltd., Tokyo, Japan.

### 2.2. Analysis of EEP

#### 2.2.1. Determination of Total Phenolic Compounds

Total polyphenols content was determined using a Folin-Ciocalteu method [58]. Phenol contents were estimated from a standard curve of gallic acid.

#### 2.2.2. HPLC-DAD Analysis

Brazilian green propolis extracts at concentration of 2.0 and 40.0 mg/mL in ethanol were used for the HPLC analysis. The concentrations of individual compounds were determined by using an external calibration curve method from the plots constructed in the range 40–300 mg/column at 308 nm (for *p*-coumaric acid and artepillin C) and 370 nm (for quercetin and kaempferol). Quercetin, kaempferol, and *p*-coumaric acid were obtained from Alexis Biochemicals (San Diego, CA, USA) and 3,5-diprenyl-4-hydroxycinnamic acid (artepillin C) was obtained from Wako Pure Chemicals (Osaka, Japan). EEP was analysed as previously reported [27].

#### 2.2.3. DPPH (1,1-Diphenyl-2-picrylhydrazyl) Radical Scavenging Activity

1,1-Diphenyl-2-picrylhydrazyl radical scavenging activity was determined using a method of Agarwal et al. [59]. Propolis extract (0.1 mL) was mixed with 2.9 mL 100 *μ*M DPPH (Sigma-Aldrich, Steinheim, Germany) in 80% aqueous methanol and stored at ambient temperature in the dark for 30 min. The decrease in absorbance of the resulting solutions was measured at 517 nm (spectrophotometer Metertek Sp-830 Medson, Paczkowo, Poland). Trolox 6-hydroxy-2,5,7,8-tetramethylchroman-2-carboxylic acid (Sigma-Aldrich, Steinheim, Germany) was used as a standard and the capacity of free radical scavenging was expressed as *μ*moles of Trolox equivalents (TEAC—Trolox equivalent antioxidant capacity). 

#### 2.2.4. ABTS Radical Cation Scavenging Activity

ABTS^•+^ scavenging activity was determined according to a procedure described by Re et al. [60]. 2,2′-Azinobis(3-ethylbenzothiazoline-6-sulfonic acid) radical cation (ABTS^•+^) was produced by the mixing of 7 *μ*M ABTS water solution and 2.45 *μ*M potassium persulfate (final concentration) and allowing the mixture to stand before use for 12–16 h in the dark at room temperature. Stock solution was diluted with PBS (pH 7.4) until an absorbance of 0.76 (±0.2) at 734 nm was reached. Each sample analysed (20 *μ*L) was mixed with 1 mL of diluted ABTS^•+^ solution and its absorption at 734 nm was measured after 6 min at 30°C (Spectrophotometer Metertek Sp-830 Medson, Paczkowo, Poland). Trolox (6-hydroxy-2,5,7,8-tetramethychroman-2-carboxylic acid) was used as a standard and the capacity of free radical scavenging was expressed as *μ*moles of Trolox equivalents (TEAC—Trolox equivalent antioxidant capacity). 

#### 2.2.5. FRAP Assay

 The FRAP (Ferric Reducing Antioxidant Power) assay developed by Benzie and Strain [61] was performed with some modification. Briefly, 2.7 mL of FRAP reagent, prepared freshly and warmed to 30°C, was mixed with water (0.27 mL) and the analysed sample (0.09 mL). The FRAP reagent was prepared by mixing 2.5 mL of a 10 mM solution of 2,4,6-tri-2-pyridyl-*s*-triazine (TPTZ) in 40 mM HCl with 2.5 mL of 20 mM FeCl_3_ *·*6H_2_O and diluting with 25 mL of 0.3 mM acetate buffer, pH 3.6. Absorbance at 593 nm was recorded after 10 min incubation of the solution at 30°C. Results were expressed as *μ*moles of Trolox equivalents (TEAC). TPTZ and Trolox were delivered from Sigma-Aldrich (Steinheim, Germany). All other chemicals were purchased from POCH (Gliwice, Poland).

#### 2.2.6. Antimicrobial Activity of EEP

The *in vitro* antibacterial activity of the EEP was determined according Xu et al. [62] with some adaptations for natural products. Seven standard bacterial strains were used: *Streptococcus mutans* ATCC 33535, *Streptococcus sanguinis* ATCC 10556, *Staphylococcus aureus* ATCC 25923, *Lactobacillus acidophilus* ATCC 4356, *Porphyromonas gingivalis* ATCC 33277, *Aggregatibacter actinomycetemcomitans* ATCC 33384, and standard yeast strain *Candida albicans* ATCC 10231 were employed in the study. These standard strains of microorganisms were acquired from the American Type Culture Collection (ATCC). The microbial standard strains suspension in tiptonic water, which contains about 3.0 × 10^7^ colony forming units (CFU), were used in this study. The EEP was dissolved in DMSO and in this examination the EEP solutions in triptonic water were used in the three final concentrations of EEP: 10 mg/L, 20 mg/L, and 50 mg/L.

The suspensions of microorganisms in triptonic water with EEP were incubated at 37°C in static conditions for 120 minutes. The triptonic water with only bacterial or yeast standard strain was tested as a blank control. The EEP (50 mg/L) in triptonic water without microorganisms and pure triptonic water were tested as a negative control. After incubation 2.0 *μ*L of each sample was seeded onto agar plate supplemented with 5% addition of the ram blood. During the experiment the surface spread plate technique was used. The agar plates were incubated at 37°C for 17 hours. Then the numbers of bacterial colonies (CFU) were counted. The following equation: ABE [%] = (*V*_*c*_ − *V*_*t*_)/*V*_*c*_ × 100 was used for investigating samples antibacterial efficacy. Numbers of viable bacterial colonies of blank control is represented by *V*_*c*_ and *V*_*t*_ stood for the test specimen.

### 2.3. Patients

The purpose of this research was assessment of effectiveness of hygienic activities conducted with use of toothpaste which contained 3% Brazilian propolis extract (EEP). The study was conducted on patients of the Dental Clinic of the Department of Conservative Dentistry with Endodontics of the Medical University of Silesia (the Academic Centre of Dentistry and Specialist Medicine in Bytom).

The research group comprised 32 adult patients (17 women and 15 men). This group was divided into two subgroups with assessment based on the preliminary evaluation of the state of their marginal periodontium:

 group I—14 patients without pathological changes within the marginal periodontium,
 group II—18 patients with danger of occurrence of periodontitis caused by dental plaque and lack of proper hygiene of the oral cavity.

Qualification for both groups was based on the interview and analysis of clinical documentation and assessment of adequate indices: API, OHI, and SBI. The Approximal Plaque Index (API) by *Lange* assesses the presence of plaque in the interdental spaces. According to that index, plaque is present or absent in the approximal spaces. The *Green *and *Vermillion* OHI index is an oral hygiene index that allows to assess debri (DI) index and calculus (CI). Periodontal status of patients from the research group was assessed with use of the *Muhlemann-Son* Sulcus Bleeding Index (SBI). Criteria of exclusion from the study: patients who did not express consent to take part in the research, juvenile patients, toothless patients, pregnant women, patients undergoing a therapy against acute systemic diseases, patients with acute periodontal diseases, and patients with recorded hypersensitivity to propolis or bee products. The research programme was approved by the Bioethics Committee of the Silesian Chamber of Medicine (resolution no. 6/2010, dated 01.03.2010).

The patients underwent three examinations: preliminary qualification and basal examination (first visit-1), a followup after 7 days (second visit-2), and an examination after 4 weeks since the beginning of the programme (third visit-3). During preliminary qualification, they were interviewed for past and current diseases, date of the last visit at the dentist's, regularity of control examinations, causes of cancelling visit arrangements, and food habits including frequency of eating carbohydrate-rich products and frequency of teeth cleaning. The patients also received professional training in hygienic procedures for the oral cavity which comprised hygiene advice, and training of correct teeth cleaning with a chosen method demonstrated on a model, training of quality control of teeth brushing procedures. The state of the oral cavity of every patient was assessed during each of the follow-up visits with use of API, OHI, and SBI indices. 

During the first, second, and third examination, the microbial material from the gingival sulcus was collected using sterile swabs. The vials were then delivered to the Department of Microbiology, Medical University of Silesia (Zabrze, Poland), within 24 hours allowing the microbial strains isolation from clinical specimens. The biological material collected for microbiological investigation was cultured on suitable medium (Columbia agar, Schaedler K3 agar, Sabouraud agar) by Biomerieux (Marcy l'Etoile, France). Aerobic bacteria were multiplied on solid medium Columbia agar, with 5% addition of ram blood, in the temperature of 37°C. Anaerobic bacteria were multiplied on solid medium Schaedler K3, with 5% addition of ram blood, in the temperature of 37°C in anaerobic conditions, with the use of GENbag anaer by Biomerieux (Marcy l'Etoile, France). Yeast fungi of the *Candida *species were multiplied on selective solid medium Sabouraud agar, in the temperature of 35°C in aerobic conditions. After they had been isolated and cultured further, each of the microorganisms was identified as regards its species, using the following set of reagents: Api 20 E, Api 20 NE, Api Candida by Biomerieux (Marcy l'Etoile, France), and ENTEROtest 24 N, NEFERMtest 24 N, STREPTOtest 24, STAPHYtest 24, and ANAEROtest 23 by Erba-Lachema (Brno, Czech Republic). 

The patients from the research groups used the following toothpaste: specimen T-toothpaste with EEP content (7 patients from group I and 9 patients from group II) or specimen G-toothpaste without EEP content (7 patients from group I and 9 patients from group II). The patients were informed about the type of hygienic preparation they were given to use (whether it contained propolis or not). The third visit ended with an interview about their subjective evaluation of received preparations. The evaluation included taste, smell, colour, and rheological properties—if they could obtain an optimal degree of foaming during tooth brushing, subjective feeling of a degree of teeth cleaning (tooth smoothness, sense of freshness in the oral cavity) assessed in a 5 grade scale.

 Data obtained from every patient were treated as confidential. Each patient had his own code initials and last two digits of their date of birth (e.g., John Doe 1960-JD/60), research group (I or II), and kind of the used toothpaste (T or G).

### 2.4. Statistical Methods

The obtained data were analysed statistically in two ways: quantitative and qualitative. Quantitative results showed values of the OHI index, whereas qualitative results were related to API and SBI indices, as well as assessment of rheological properties of tested preparations and subjective evaluation of the patients' impressions of the degree of comfort when using the products. Both parametric and nonparametric tests were used for statistical verification of the assumed research hypotheses. Use of the first group of tests requires verification of assumptions made for normality and homogeneity of variance of the data, which was verified with use of the *Shapiro-Wilk* test (normality) and the *Lavene* test (homogeneity of variance). The ANOVA method was used in order to statistically assess the dynamics of changes of the API index which occurred due to the effect of the preparations. However, due to the fact that the data was a set of repeated measurements, the *Wilks *lambda test was used as a multidimensional equivalent of the *F*-test. As the null hypothesis of equality of means was rejected, the *Tukey* range test was used to compare individual means for each of the groups. Student's *t*-test was used to assess influence of the toothpaste and gel on the quantitative parameters, provided that the assumption of variance homogeneity was fulfilled. If not, a *t*-test with separate variance estimate (called the *Cochran-Cox* test) was utilized. The statistical analysis of qualitative parameters was based on nonparametric tests. More than two dependent samples were compared with use of the *Friedman *ANOVA test, while in the case of just two samples, the *Wilcoxon* signed rank test was used. All tests were conducted for the significance level of *λ* = 0.05 and with use of Statistica v.8 software (Statsoft, Kraków, Poland).

## 3. Results

 Brazilian green propolis contains various chemical components. The identification of individual compounds in EEP was performed on the basis of their retention times. Thus, the main flavonoid compounds present in EEP are kaempferol and quercetin. We have also detected other compounds, cinnamic acid derivatives such as p-coumaric acid and artepillin C. The quantification is shown in Table 1.

The aim of this work was to investigate the antioxidant property of EEP.

These results indicate that antioxidant activities of Brazilian green EEP are as follows:

 FRAP: 2694.87 ± 100.60 micromoles Trolox equivalents per gram of extract (*μ*moles Trolox/g),
 DPPH: 1230.07 ± 135.55 *μ*moles Trolox/g,
 ABTS: 1223.06 ± 137.40 *μ*moles Trolox/g.

These studies investigated EEP activity against bacteria (*Streptococcus mutans* ATCC 33535, *Staphylococcus aureus* ATCC 25923, *Lactobacillus acidophilus* ATCC 4346, *Aggregatibacter actinomycetemcomitans* ATCC 33384, *Streptococcus sanguinis* ATCC 10556, *Porphyromonas gingivalis* ATCC 33277), and *Candida albicans* ATCC 10231.

**Figure 1:**
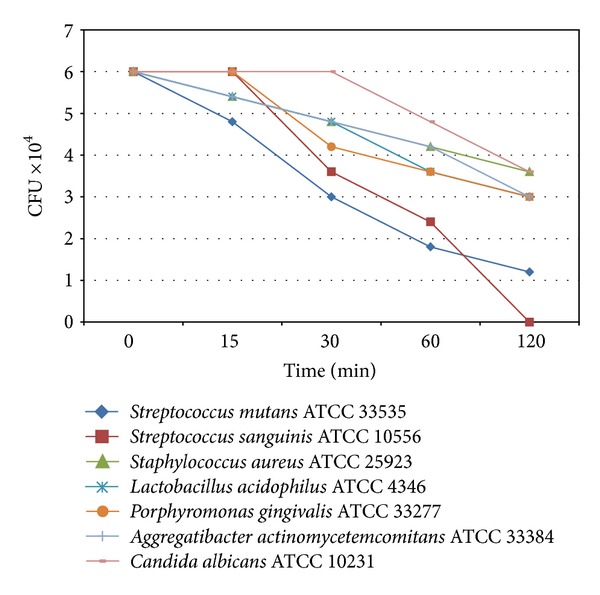
The kill curve of standard microorganisms strains, which indicated the time-dependent antimicrobial activity of the EEP (used concentration of EEP was 50 mg/L).

Results of microbial susceptibility of EEP are summarized in Figure 1. EEP displayed patent antimicrobial activity against Gram-positive bacteria as well as the yeast. Results obtained clearly indicated time-dependent microbial action of EEP at 50 mg/L concentration.

**Figure 2:**
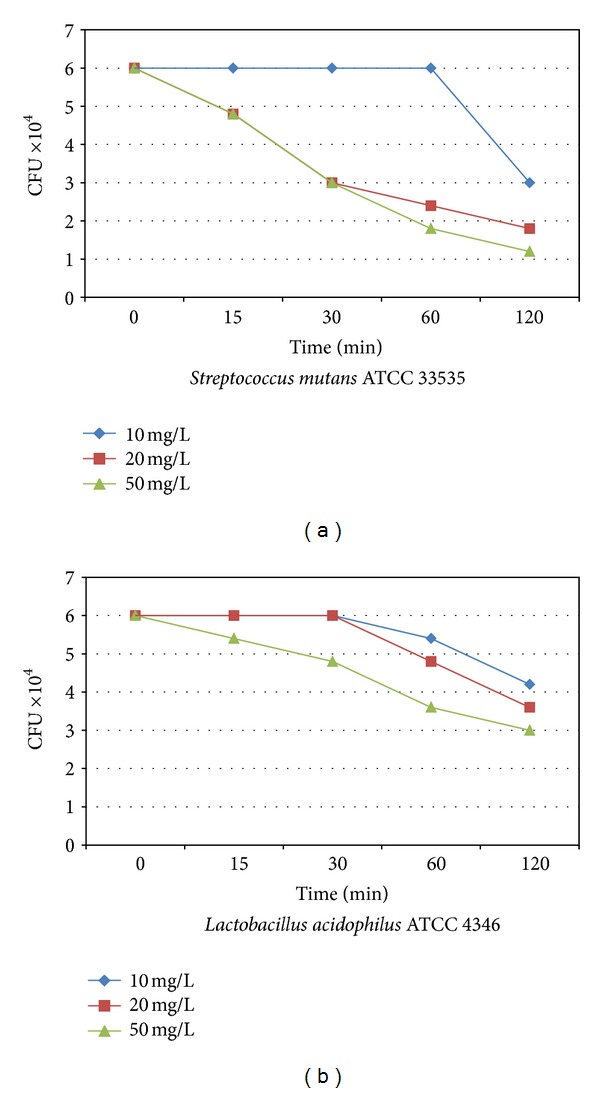
The kill curve of *Streptococcus mutans* (a) and *Lactobacillus acidophilus* (b) standard strains, which indicated the time-dependent antimicrobial activity of the EEP (used concentrations of EEP were: 10 mg/L, 20 mg/L and 50 mg/L).

The EEP used in this study no activity was observed against *E. coli*. The major activity of the extract was found against *S. mutans* and *L. acidophilus* (Figure 2).

 In case of the OHI index (*Green* and *Vermillion's* Oral Hygiene Index) and patients of Groups I and II who used T or G toothpastes, the value decreased so much that the median (average value) after 4 weeks was statistically different from the value during the first visit (*P* = 0.0679) (Table 2). It suggests that in the case of patients who were not diagnosed with periodontal diseases but only with some tendencies due to lack of hygiene, the fact of participating in the research programme and the necessity of using hygienic preparations and a toothbrush alone could have played a significant role, irrespective of the presence or absence of a propolis additive. However, it may suggest that the choice of therapeutic product such as propolis may have a significant influence on elimination of hygienic negligence in case of healthy patients or patients with minor problems (Table 2).

 The *Lange's* API index values in case of those groups of patients who were to start using the T or G toothpaste varied from fully correct values during the first visit. Results of evaluation in case of the patients from Group I yielded a range of values that allowed the qualification of those patients in a range described as *“quite good hygiene*,*”* while during a follow-up visit that took place 7 days after the initial examination, their results changed the qualification to a range described as *“optimal hygiene*.*”* Moreover, during the third visit, the qualification changed back to the range described as *“quite good hygiene*.*”* The patient Group II received a qualification *“average hygiene”* during the first visit and remained the same during the follow-up examinations.

 In case of the patients from Group I, who used the T or G toothpaste, the values of SBI index (*Muhlemann-Son's* Sulcus Bleeding Index) qualified them to be included in the range described as *“gingiva normal, no bleeding”* and *“bleeding after probing without changes in the shape and colour”* during the initial examination. During the third examination, all patients qualified to the range described as *“gingiva normal, no bleeding”*. However, that tendency did not prove to be statistically significant (Table 4). In case of the patients from Group II, who used the T or G toothpaste, the initial qualification was *“bleeding after probing without changes in the shape and colour”* and no statistical significance was observed during the third examination (Table 3). 

The assessment of rheological properties of researched preparations as well as the patients' impressions of the degree of comfort when using the preparations (including taste, smell, colour, optimal degree of foaming during tooth brushing, and subjective feeling of a degree of tooth cleaning, such as tooth smoothness and sense of freshness in the oral cavity) showed statistical significance in case of the T and G toothpaste in such parameters as taste, colour, and foaming ability (Table 4).

 The differences in the type and number of the microorganisms strains and species, isolated from gingival sulcus of patients from Group I and II who used the T or G toothpastes, were presented in Tables 5, 6, and 7.

## 4. Discussion

Alternative or complementary medicine is a collection of concepts, means, and techniques based on natural forces, nature, and human powers. Some branches of complementary medicine have their origins in many hundred-year-old traditions (e.g., acupuncture). Current trends show a return to natural medicine and treatment methods, also due to the fact that patients are worried about preventive means of fighting many diseases and illnesses. Bee products are an example of such means that have been widely and commonly used for many years and that can improve our health as well as play an important role in prevention of illnesses. The therapeutic potential of honey has recently been reviewed. Other bee products, royal jelly and propolis, have also been widely used in *“folklore medicine”* for centuries. As popular folk medicine, propolis is alleged to exhibit a broad spectrum of activities including antibiotic, anti-inflammatory, and tumour growth arrest; some of the observed biological activities may be traced to identified chemical constituents such as caffeic acid which is antimicrobial and anti-inflammatory [5, 11, 18, 63].

Owing to discoveries in the field of chemical composition and confirmed usefulness of those products by both laboratory and clinical research, a term of “apitherapy” was coined. It is a distinguished method of treatment based on use of products which were collected, processed, or produced by bees. Such products include the following: pollen—as a product collected by bees, propolis, nectar honey, bee bread—products collected and processed by bees, royal jelly, bee venom, beeswax—products secreted by bees [18].

Therapeutic properties of propolis have been used in dentistry for many years, focused on the four most well-known properties of that substance: regenerative, antibacterial, anaesthetic, and stimulating the immune system. Propolis has been shown to exhibit very good antimicrobial activity against a range of oral bacteria [12]. The effect of propolis on growth and glucosyltransferase activity of *Streptococcus sobrinus*, *Streptococcus mutans,* and *Streptococcus circuits* was observed *in vitro* and *in vivo* [64] and it was found that the in soluble glycan synthesis and glucosyltransferase activity were inhibited by multiple actions of propolis. Koru et al. (2007) studied the antibacterial action against certain anaerobic oral pathogens and found it to be very effective against *Peptostreptococcus anaerobius*, *Lactobacillus acidophilus*, *Actinomyces naeslundii*, *Prevotella oralis. Prevotella melaninogenica, Porphyromonas gingivalis, Fusobacterium nucleatum, *and* Veillonella parvula.* They concluded that the antibacterial property of propolis is due to the presence of flavonoids and aromatic compounds such as cinnamic acid [20]. Agarwal et al. suggest that Chinese propolis has potent antimicrobial activity against this periodontopathogens, suggesting its possible use as a natural alternative to the widely used antibiotics for periodontal therapy [59].

Inflammatory stimulation by periodontal pathogens increases the production of crevicular fluid and induces the chemotaxis of neutrophils, which in order to inactivate periodontal pathogens, releases singlet oxygen and hypochlorous acid into the crevicular fluid. The consequent oxidative stress is countered by the antioxidant activity of ascorbate albumin and urate present in the crevicular fluid and derived from plasma. However, this local oxidative stress may be increased by external factor or systemic conditions, such as smoking, diabetes, obesity, and metabolic syndrome. When there is a disequilibrium between oxidative stress and antioxidant activity, periodontal tissue destruction may appear. These observations suggest that antioxidant rich diets might inhibit periodontal disease development and progression, particularly in subjects exposed to environmental and dietary sources of oxidative stress [65].

Studies on phenolic compounds and antioxidant activity of propolis have been reported recently [49, 66, 67]. 

The amounts of total polyphenol in our propolis extract is 245.52 ± 8.29 mg/g (as gallic acid equivalents). According to the literature data, polyphenol content in propolis extract varies from 85 to 283 mg per g of ethanol extract [49].

It is also well known that propolis shows very high antioxidant activity [49, 66, 67]. In the present study the antioxidant activity of propolis extract has been evaluated by using three different assays: FRAP (Ferric Reducing Antioxidant Power) and methods for the stable ABTS^•+^ and DPPH^•^. The examined propolis extract shows very high activities in all systems. TEAC value (*μ*moles Trolox/g) reaches: 2694.87 ± 100.60, 1230.07 ± 135.55, and 1223.06 ± 137.40 for FRAP, ABTS, and DPPH methods, respectively. The synthetic nitrogen-centred DPPH radical is not biologically relevant, but DPPH assay is often used to evaluate the ability of antioxidant to scavenge free radicals that are known to be a major factor in biological damages caused by oxidative stress [29]. Our results are in agreement with the literature data [49, 63, 64] although expressed in different ways.

The regenerative features of propolis were observed by Stojko et al. in animals [68]. Soft tissue and cartilage wounds of dogs granulate quickly if the ethanol extract of propolis (EEP) or a water solution of propolis was applied. Moreover, significant acceleration of cartilage and perichondrial primordia was found during the histopathological and radiological examinations. Cytochemical research allowed noticing increased mitochondrial activity of the letrozole reductase, which indicates increased cell metabolism. Therapeutic properties of propolis can be confirmed with its effectiveness in the periodontal diseases, as it mitigates the course of alveolar osteitis [68]. Its anaesthetic properties are used in dentin hypersensitivity, electrocoagulation of hypertrophic interdental papillae, and minor surgical procedures. There are also reports on research tests with use of propolis in treatment of the pulp and caries. Propolis is also used in a form of gel and rubbing ointment, 10-percent ethanol solution for brushing, and a 0.2-percent ethanol-water solution for mouthwashing used three times a day in the treatment of periodontal diseases, ulcerative gingivitis, chronic and recurrent aphthous ulcers, desquamative cheilitis and bullous, and ulcerative forms of lichen planus. Treatment can be supported with chewing three times a day for half an hour of a 3-gram lump of propolis, while people with dentures can sprinkle them with a powdered form. Propolis extracts prove to be highly effective in soothing postextraction pain and treatment of alveolar osteitis. Repeated intra-alveolar application of a seton soaked with EEP takes away the pain and accelerates wound healing [69]. Further research on applications of the etanol propolis extract demonstrate it in dentistry in three different forms *in substantia, *in a 3-percent ethanol-glycerine solution and as a 3-percent ointment on an *Eucerinum anhydricum* base. EEP was rubbed into the hard tooth tissue during preparation of a cavity in order to anaesthetise it. The anaesthetic effect was obtained after 3 to 4 minutes of rubbing the preparation into the walls and bottom of the cavity. The analysis of influence of EEP in dependence of the depth of the cavity demonstrated a slightly higher percentage of cases in which anaesthesia was complete in case of average caries or even deep caries than in case of surface caries. During treatment of hypersensitivity of the dentin, dried and isolated from saliva, teeth were rubbed with EEP for a period of 3 to 4 minutes, every 2 to 3 days. In case of caries, prepared cavity was washed with an EEP solution which created a thin, organic film that blocked the exit of the dentin canals after the vapour fraction disappeared. In case of pulp treatment, not only antiseptic properties were observed but also long-term treatment benefits could be noted. In some cases, for example, for direct covering of the pulp, EEP *in substantia* was used instead of a ready solution, similarly to the cases of pulpotomy [70].

 Results of the presented research show effectiveness of hygienic preparations with 3% content of EEP in both groups of patients: without the pathological changes of periodontium and in the case of patients endangered with occurrence of gingivitis caused by dental plaque. When using propolis, particular attention should be paid to its side effects, particularly possible occurrence of allergic reactions. Some authors claim that the frequency of allergic cases constitutes 0.25% of all propolis applications, and others confirm this number to be as large as 0.8% of cases in which oedema of mucous membranes or conjunctivas, as well as hypotension or cardiac dysrhythmia may occur [69].

## 5. Conclusion

Hygienic preparations with 3% content of Brazylian ethanol extract of green propolis (EEP) efficiently support removal of dental plaque and improve the state of marginal periodontium.

## Figures and Tables

**Table 1:** Quantification of main compounds in EEP^a^.

	mg/g EEP
Quercetin	0.48 ± 0.02
Kaempferol	3.20 ± 0.10
P-Coumaric acid	15.00 ± 0.50
Artepillin C	58.90 ± 2.60

^a^Total compounds content are expressed as milligrams in 1 g of EEP. All data are the mean ± SD of three independent determinations. Total polyphenols compounds in EEP is 254.52 ± 8.29 mg/g.

**Table 2:** Median [min–max] for OHI index assessment of Group I and II patients who used the T or G toothpastes.

Group	Specimen	Examination				Wilcoxon's signed rank test		
		First (1)	Second (2)	Third (3)	ANOVA Friedma (*P*)	Comparable group pairs		
						(1) : (2)	(1) : (3)	(2) : (3)
I	T	2 [1-2]	1 [1-2]	1 [1-1]	0.0498*	0.4795	*0.0679 *	0.4795
	G	1 [0–2]	1 [0–2]	1 [0–2]	0.6065			

II	T	2 [1–3]	2 [1–3]	2 [1–3]	0.0388*	0.108	*0.0679 *	0.5837
	G	1 [1-2]	1 [1-1]	1 [1-2]	0.0366*	0.108	*0.0679 *	0.4795

*P*—test probability.

*High level of significance.

**Table 3:** Median [min–max] for SBI index assessment of Group I and II patients who used the T or G toothpaste.

Group	Specimen	Examination			ANOVA Fridman (*P*)
		First (1)	Second (2)	Third (3)	
I	T	0 [0-1]	1 [0-1]	0 [0-1]	0.4724
	G	1 [0-1]	0 [0-1]	0 [0-1]	0.3679

II	T	1 [1-2]	1 [0-1]	1 [0-1]	0.2636
	G	1 [1-2]	1 [1-1]	1 [1-1]	0.1354

*P*—test probability.

**Table 4:** Median [min–max] for preparation properties assessment of Group I and II of patients.

Property	Specimen		Mann-Whitney's *U*-test (*P*)
	T	G	
Taste	3 [2–4]	4 [2–5]	0.0332*
Smell	3 [2–4]	4 [2–5]	0.1822
Colour	2 [1–5]	4 [2–5]	0.00001*
Foaming	2 [1–4]	3 [1–5]	0.0198*
Cleaning	3 [1–5]	4 [1–5]	0.2336

Descriptive marking scale for each property of used preparation was limited by the following ranges: 1—unsatisfactory, 2—satisfactory, 3—average, 4—good, 5—very good.

*High level of significance.

**Table 5:** The number of microorganisms strains isolated from gingival sulcus of Group I patients, who used toothpaste with or without propolis.

Species of microorganisms	Group I—patients, who used					
	Toothpaste with propolis (specimen T)			Toothpaste without propolis (specimen G)		
	Examination			Examination		
	First (1)	Second (2)	Third (3)	First (1)	Second (2)	Third (3)
Gram-positive cocci:						
*Streptococcus canis *	—	—	1	—	1	1
*Streptococcus constellatus *	—	—	—	—	—	1
*Streptococcus intermedius *	—	—	4	2	2	2
*Streptococcus mitis *	1	1	1	2	3	3
*Streptococcus salivarius *	1	4	—	2	2	1
*Streptococcus sanguinis *	5	3	—	2	—	1
*Streptococcus parasanguinis *	—	—	—	1	—	—
*Staphylococcus aureus *MSSA	—	—	—	—	1	—
*Staphylococcus capitis *	1	—	—	—	—	—
*Staphylococcus epidermidis *MSCNS	—	1	—	1	—	—
*Staphylococcus hominis *	—	—	1	—	—	1
*Staphylococcus xylosus *	—	—	—	—	1	—
Gram-negative cocci:						
* Veillonella parvula *	1	—	2	—	1	—
Gram-positive rods:						
* Atopobium minutum *	—	1	—	—	—	—
* Bifidobacterium breve *	1	—	—	—	—	—
* Bifidobacterium infantis *	—	—	—	—	—	1
* Bifidobacterium longum *	—	—	—	1	—	—
* Clostridium baratii *	—	—	—	1	—	—
* Clostridium butyricum *	—	—	—	1	—	—
* Clostridium perfringens *	—	—	—	—	1	—
* Clostridium ramosum *	1	—	—	—	—	—
* Clostridium tertium *	—	—	—	—	1	—
* Propionibacterium acnes *	1	—	—	—	—	—
* Actinomyces israelii *	—	—	—	1	—	—
* Actinomyces viscosus *	—	—	—	—	1	—
Gram-negative rods:						
* Bacteroides ureolyticus *	1	—	—	—	—	—
* Campylobacter gracilis *	—	1	1	—	—	—
* Escherichia coli *	—	—	—	1	—	—
Yeast fungi:						
* Candida albicans *	—	—	—	1	—	—

Number of microorganisms strains (altogether)	**13**	**11**	**10**	**16**	**14**	**11**
		**(34)**			**(41)**	

**Table 6:** The number of microorganisms strains isolated from gingival sulcus of Group II patients, who used toothpaste with or without propolis.

Species of microorganisms	Group II—patients, who used					
	Toothpaste with propolis (specimen T)			Toothpaste without propolis (specimen G)		
	Examination			Examination		
	First (1)	Second (2)	Third (3)	First (1)	Second (2)	Third (3)
Gram-positive cocci:						
* Streptococcus canis *	—	—	1	1	1	1
* Streptococcus intermedius *	3	1	3	3	6	1
* Streptococcus mitis *	4	5	1	3	4	2
* Streptococcus salivarius *	1	1	2	—	—	1
* Streptococcus sanguinis *	1	3	2	1	—	2
* Streptococcus parasanguinis *	1	—	1	—	—	—
* Streptococcus vestibularis *	—	—	—	1	1	—
* Enterococcus avium *	1	—	—	1	—	1
* Enterococcus faecalis *	—	—	—	1	1	1
* Gemella morbillorum *	—	—	1	—	—	1
* Peptococcus niger *	—	—	—	—	—	1
* Staphylococcus aureus *MSSA	—	1	—	—	—	—
* Staphylococcus epidermidis *MSCNS	1	—	1	2	—	—
* Staphylococcus haemolyticus *MSCNS	—	—	—	—	1	—
* Staphylococcus hominis *	—	—	—	—	1	—
* Staphylococcus werneri *	—	—	—	—	1	—
Gram-negative cocci:						
* Veillonella parvula *	—	1	2	—	—	1
Gram-positive rods:						
* Atopobium parvulum *	—	—	—	—	—	1
* Bifidobacterium adolescentis *	1	—	—	—	—	—
* Bifidobacterium dentium *	—	2	—	1	—	—
* Bifidobacterium infantis *	—	—	—	—	—	1
* Clostridium baratii *	—	—	—	1	—	—
* Clostridium novyi *	—	—	—	1	—	—
* Clostridium ramosum *	—	—	—	1	—	—
* Propionibacterium propionicus *	—	—	—	—	1	—
* Actinomyces israelii *	—	—	—	—	—	1
Gram-negative rods:						
* Acinetobacter haemolyticus *	—	—	—	—	—	1
* Bacteroides ureolyticus *	—	—	—	—	—	1
* Campylobacter gracilis *	—	1	—	—	—	—
* Edwardsiella hoshinae *	—	—	—	—	—	1
* Mitsuokella multiacidus *	—	—	—	—	—	1
* Serratia liquefaciens *	1	—	—	—	—	1
Yeast fungi:						
* Candida albicans *	—	—	1	3	—	—

Number of microorganisms strains (altogether)	**14**	**15**	**15**	**20**	**17**	**20**
		**(44)**			**(57)**	

**Table 7:** The number of the microorganisms species isolated from gingival sulcus of patients from Group I and Group II, who used the toothpaste with propolis (specimen T) or without propolis (specimen G).

Group	Specimen	Examination		
		First (1)	Second (2)	Third (3)
I	T	9	6	6
	G	12	10	8

II	T	9	8	10
	G	13	9	18
